# Phase-selective entrainment of nonlinear oscillator ensembles

**DOI:** 10.1038/ncomms10788

**Published:** 2016-03-18

**Authors:** Anatoly Zlotnik, Raphael Nagao, István Z. Kiss, Jr-Shin Li

**Affiliations:** 1 Center for Nonlinear Studies, MS B258, Los Alamos National Laboratory, Los Alamos, New Mexico 87545, USA; 2 Department of Chemistry, Saint Louis University, 3501 Laclede Ave., St Louis, Missouri 63103, USA; 3 Department of Electrical and Systems Engineering, Washington University in St Louis, CB 1042, 1 Brookings Drive, St Louis, Missouri 63130, USA

## Abstract

The ability to organize and finely manipulate the hierarchy and timing of dynamic processes is important for understanding and influencing brain functions, sleep and metabolic cycles, and many other natural phenomena. However, establishing spatiotemporal structures in biological oscillator ensembles is a challenging task that requires controlling large collections of complex nonlinear dynamical units. In this report, we present a method to design entrainment signals that create stable phase patterns in ensembles of heterogeneous nonlinear oscillators without using state feedback information. We demonstrate the approach using experiments with electrochemical reactions on multielectrode arrays, in which we selectively assign ensemble subgroups into spatiotemporal patterns with multiple phase clusters. The experimentally confirmed mechanism elucidates the connection between the phases and natural frequencies of a collection of dynamical elements, the spatial and temporal information that is encoded within this ensemble, and how external signals can be used to retrieve this information.

## Abstract (web summary)

Organizing and manipulating dynamic processes is important to understand and influence many natural phenomena. Here, the authors present a method to design entrainment signals that create stable phase patterns in heterogeneous nonlinear oscillators, and verify it in electrochemical reactions.

## Graphical abstract

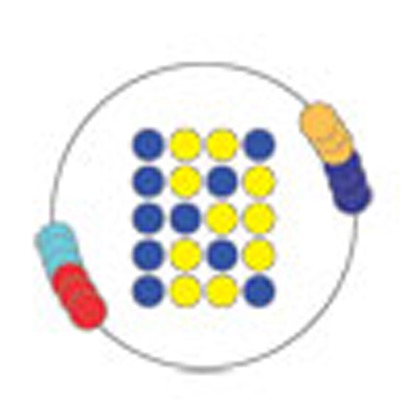

Complex interactions among nonlinear periodic phenomena emerge in many natural and engineered systems^1^,^2^. Numerous instances appear in chemical reactions^3^,^4^ and biological systems^5^,^6^, which exhibit endogenous and emergent multiscale oscillations^7^. There is significant interest in characterizing synchronization in oscillators interconnected in networks^8^,^9^, which is especially important for understanding the highly complex dynamics of man-made systems such as electric power grids^10^, and elucidating the functions of neural systems^11^,^12^,^13^. Understanding entrainment of oscillating systems to an exogenous forcing signal is crucial to modelling circadian timekeeping^14^, dynamic neural regulation^15^ and for the design of synchronizing or desynchronizing treatments of cardiac arrhythmias^16^, Parkinson's disease^17^, epilepsy^18^ and movement disorders^19^.

Even the simplest models of interacting oscillators can exhibit highly complex behaviour^20^, and individual oscillating units may themselves possess complicated dynamics^21^. These factors are aggravated in practice by model and parameter uncertainty and the impracticality of obtaining feedback information, such as for *in vivo* biological applications, and pose challenges to manipulating or controlling oscillating ensembles. Such tasks require tractable yet accurate simplifications of the complex dynamic interactions involved, and demand suitable mathematical approaches that characterize ensemble-level properties while withstanding experimental uncertainties.

Control-theoretic techniques have been applied to control a single oscillator^22^,^23^,^24^. In contrast, finely manipulating individual subsystems in underactuated ensembles, such as thousands of neurons in the brain affected by one electrode, rather than activating them homogeneously remains a fundamental challenge. Synchronization has been engineered in collections of oscillators using feedback^25^,^26^,^27^, or tuning coupling strengths^4^,^28^,^29^. Such approaches require certain coupling structures, exact model specification, state feedback information, or precise knowledge of initial conditions, but still are not able to produce a prescribed phase pattern corresponding to frequency clusters of the oscillators.

Versatile open-loop control techniques were developed for simultaneous control of ensembles of quantum spin systems, which motivated the field of ensemble control^30^. Inspired by selective pulse design in nuclear magnetic resonance (NMR)^31^, which enabled revolutionary applications including functional magnetic resonance imaging (fMRI), we develop a method for selectively manipulating the subunits of oscillator ensembles using periodic inputs that are robust to parameter uncertainty and disturbances. Specifically, we exploit the slight heterogeneity and high nonlinearity of an ensemble of structurally similar oscillators far past the Hopf bifurcation, rather than relying on a known coupling structure, state feedback or initial condition information.

In this manuscript, we present a methodology for constructing weak, globally applied, open-loop control inputs that synchronize a collection of structurally similar yet heterogeneous nonlinear oscillators while selectively assigning their relative phases on the periodic orbit. Using the technique, the synchronization structure of an oscillating ensemble can be manipulated among diverse phase patterns, seen in relative positions on the limit cycle. Our theory is developed specifically to overcome the challenges of experimental implementation when feedback information is unavailable, initial conditions are unknown and the oscillators are subject to uncertainty in subsystem parameters and stochastic disturbances. The control inputs create and maintain such phase patterns when the coupling between oscillators is weak, while preserving the intrinsic nature of the ensemble to enable nondestructive application to fragile biological and chemical systems. The dynamics of the oscillators may be arbitrary, as long as all are structurally similar and exhibit sufficient nonlinear relaxation for the control design to be realizable. A coherent structure may be established and robustly maintained indefinitely by a single periodic waveform, which can be altered to switch between patterns. We demonstrate the theoretical methodology in practice with experiments to control complex electrochemical ensembles whose dynamics are variable and unknown, and for which state information is unavailable^26^,^32^.

## Results

### Phase model approximation

We approximate the effect of inputs on periodic dynamical systems using phase models^33^, which can be computed for systems with known dynamics^34^ or experimentally inferred in practice when the dynamics are unknown^35^. These models are used to characterize circadian cycles^36^, cardiac rhythms^16^ and phenomena in neural and chemical systems^11^,^37^, and their simplicity has enabled control design for neuron models^22^ given initial conditions and exact parameters. Control techniques have recently been developed for individual nonlinear oscillators and finite collections described by phase models that require exactly known initial conditions, parameters, and dynamics^22^,^38^,^39^. Many studies on synchronization focus on the network structure of couplings between oscillators, and the nonlinearity in the phase response of each unit is simplified to sinusoidal couplings with its neighbours^9^. However, for the manipulation and desynchronization of electrochemical and neural systems^11^,^40^,^41^, complex, hierarchical interactions must be established or broken in large collections of nonlinear systems. The dynamics, parameters, and interconnections of these systems are typically problematic to infer, may be noisy, variable, or uncertain, and state observations may be incomplete or unavailable. Such conditions elude tractable formulation, and require an approach where synchronization properties of the systems, that is, asymptotic phase structure, are manipulated rather than steering the system states directly^7^,^42^.

### Entrainment of an ensemble

**Figure 1:**
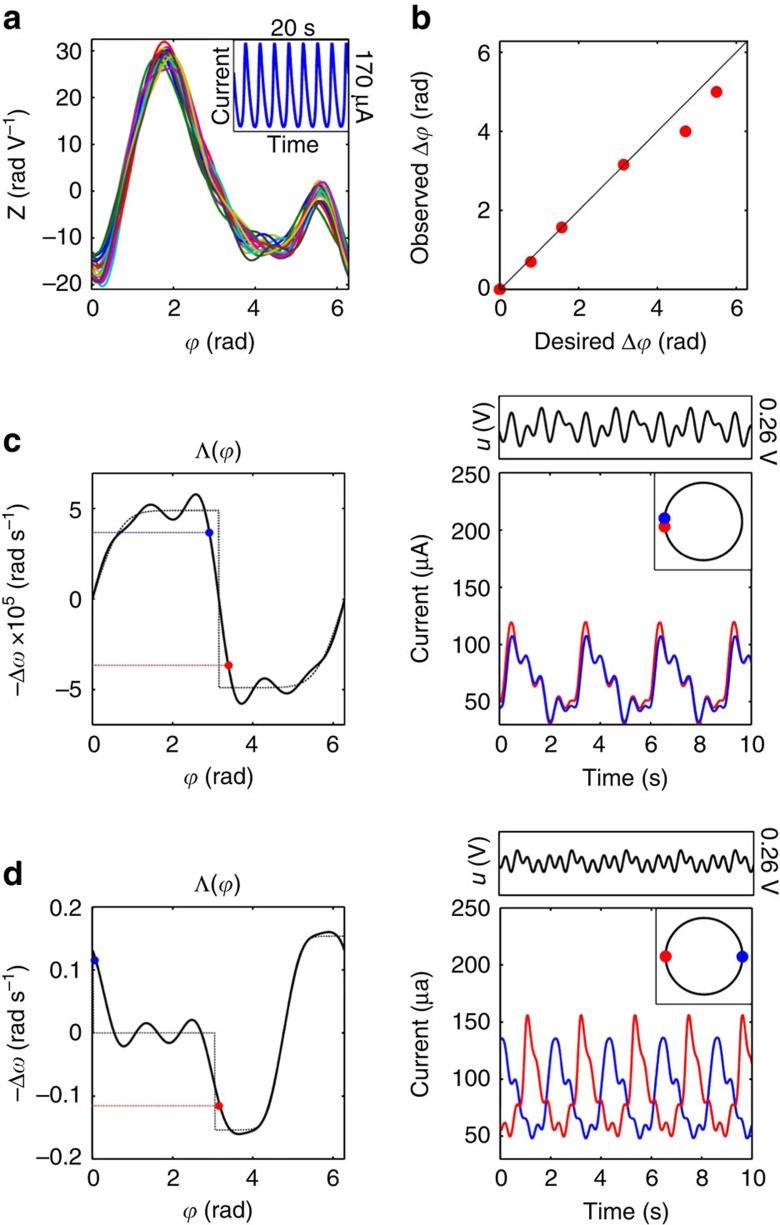
Tuning the phase difference between two electrochemical oscillators. (**a**) PRCs measured simultaneously for 20 working electrodes, and observed current oscillations (inset). (**b**) Designed versus experimentally measured phase difference and fit (dotted line). The left panels in **c** and **d** show how phase assignment is constructed using an ideal interaction function (dotted line), and the respective best achievable approximation (solid line) for two nonidentical oscillators. The right panel shows the time-series of the entrained oscillators and the periodic control signal (above), and the entrained oscillator phases on the unit circle (inset). (**c**) In-phase phase assignment: 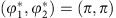, with natural frequencies (*ω*_1_, *ω*_2_)=(0.330, 0.348) Hz shown in (blue,red) with Ω=0.339 Hz. (**d**) Anti-phase phase assignment: 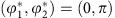 and natural frequencies (*ω*_1_, *ω*_2_)=(0.443, 0.480) Hz in (blue,red), with Ω=0.462 Hz.

Our method relies on entrainment, which refers to the dynamic synchronization of oscillating systems to periodic inputs. Each system in an ensemble of *N* structurally similar units exhibits endogenous oscillation along an attractive periodic orbit with period *T*_*j*_, and is represented by the Winfree phase model


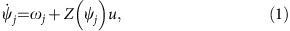


where *ω*_*j*_=2*π*/*T*_*j*_ is the natural frequency and *Z* is the phase response curve (PRC), which quantifies how a weak perturbation *u* advances or delays the phase 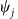 (refs 2, 33). A value of 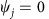 (equivalently 2*π*) corresponds to a measurement of the *j*th system reaching its maximum. More details on phase coordinate transformation are given in Supplementary Note 1. We demonstrate our phase-selective entrainment techniques using experiments in which nickel electrodes undergo anodic dissolution in sulfuric acid and exhibit electrochemical oscillations^32^, for which the experimental apparatus is described in section ‘Experimental apparatus'. Before these experiments, the PRCs of the ensemble elements, which are shown in Fig. 1a, were estimated and averaged for use as the nominal PRC in equation (1). This was done using a pulse perturbation procedure for system identification that was previously used for electrochemical oscillators^43^, and is described in Supplementary Note 2.

To synchronize oscillation of ensemble elements, each subsystem receives the same weak, periodic forcing input of frequency Ω of the form *u*(*t*)=*v*(Ω*t*), where *v* is 2*π*-periodic. When the forcing frequency is near the natural frequencies in the ensemble, averaging theory^44^ states that the mean difference 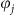 between each phase 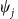 and the forcing phase *θ*=Ω*t* follows the time-independent dynamic equation


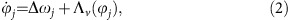


where Δ*ω*_*j*_=*ω*_*j*_−Ω is called the frequency detuning, and


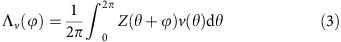


is a 2*π*-periodic interaction function that characterizes the average effect of the periodic input on the oscillation phase^43^. Such ergodic averaging is discussed in more detail in Supplementary Note 3 and illustrated in Supplementary Fig. 1. If equation (2) has a unique attractive fixed point 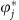 that satisfies 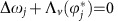, then the phase of the *j*th oscillator becomes entrained to the forcing phase *θ* with an average offset of 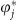. This analysis is widely applied to determine the interaction function resulting from a measured PRC and an input waveform, and equation (2) is used to infer the entrained system's stability^27^. We reverse this approach by choosing the desired asymptotic behaviour, constructing a suitable interaction function and using the PRC to obtain the input by circular deconvolution of equation (3).

### Interaction functions for phase selection

We design the input *v*(Ω*t*) so that each system in equation (1) is entrained to a forcing frequency Ω, e.g., the mean of natural frequencies *ω*_1_<*ω*_2_<…<*ω*_*N*_, and such that the *j*th oscillator cycles its orbit with a phase offset of 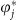 relative to the forcing phase θ. The set of pairs 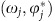 constitutes a pattern for selective entrainment. We require that 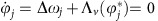 eventually holds for each oscillator, that is, equation (2) has an attractive fixed point at 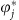 for all *j* at which the slope of the interaction function Λ_*v*_ is negative^43^. The function that best satisfies these ideal conditions has steep decreases at phase values 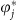 where entrainment must occur, and crosses (from above) horizontal lines at frequency detuning values −Δ*ω*_*j*_. This creates the desired attractive fixed points for equation (2). Because the interaction function is periodic, it must then increase so that Λ_*v*_(2*π*)=Λ_*v*_(0) holds. Crossings of −Δ*ω*_*j*_ from below are unstable fixed points, and do not affect convergence.

The concept is illustrated in Fig. 1, which describes an experiment where a phase difference 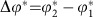 is assigned between two entrained oscillators. In Fig. 1c we desire in-phase synchronization at phase offsets of 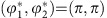, so the ideal interaction function has one steep decrease at 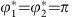 that intersects horizontal lines through −Δ*ω*_1_ (blue) and −Δ*ω*_2_ (red) at π radians. Figure 1d illustrates anti-phase synchronization with 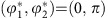, where the interaction function has two steep decreases at 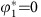 and 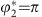 that intersect horizontal lines at −Δ*ω*_1_ (blue) and −Δ*ω*_2_ (red), respectively. The best achievable interaction function (solid line) and the PRC estimate yield the input from equation (3). The right columns of Fig. 1c,d show the observed current of two oscillators entrained in in-phase and anti-phase arrangements by the input waveform (shown above). These configurations are achieved regardless of initial oscillator phases, because the interaction function crosses the line −Δ*ω*_*j*_ only once from above, so each system has a globally attractive fixed point. For the electrochemical system, phase differences in nearly the entire 0 to 2*π* region are achievable, with small deviations as 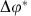 approaches 2*π*, as seen in Fig. 1b.

### Separation of oscillator ensembles into phase clusters

**Figure 2:**
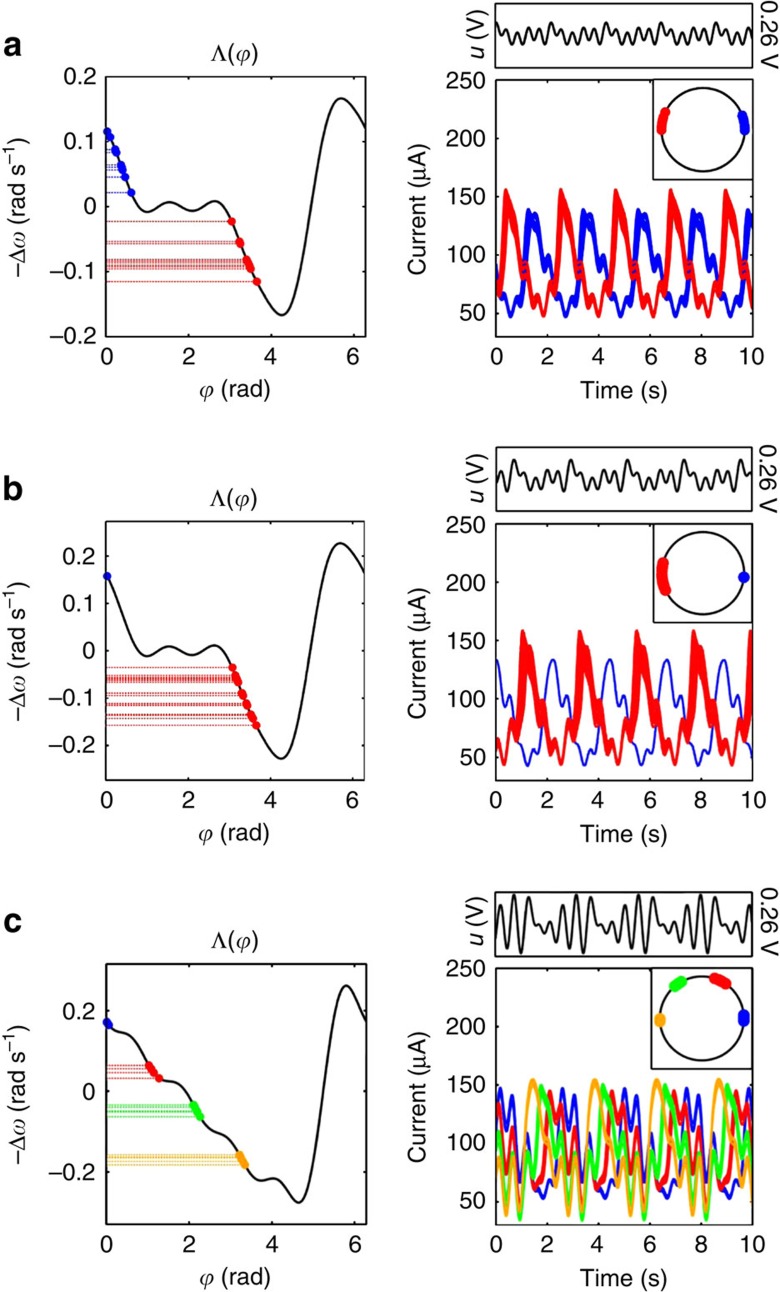
Phase assignment for 20 electrochemical oscillators. The first column shows the frequency detunings and the intersections with the interaction function at the phase offsets 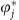. The second column represents the entrained time-series with the respective periodic waveform of the control signal (above) and a plot of the phase offsets on the unit circle (inset). Phase assignment for: (**a**): (*N*_1_, *N*_2_)=(10, 10) clusters; cluster 1 (blue): 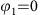 and *ω*_1_=0.450 Hz; cluster 2 (red): 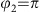 and *ω*_2_=0.471 Hz; forcing at Ω=0.463 Hz. (**b**): (*N*_1_, *N*_2_)=(1, 19) clusters; one electrode (blue): 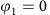 and *ω*_1_=0.419 Hz, cluster 2 (red): 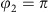 and *ω*_2_=0.454 Hz; forcing at Ω=0.450 Hz. (**c**): (*N*_1_, *N*_2_, *N*_3_, *N*_4_)=(4, 4, 4, 4) clusters; cluster 1 (blue): 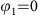 and *ω*_1_=0.386 Hz, cluster 2 (red): 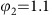 rad and *ω*_2_=0.404 Hz; cluster 3 (green): 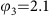 rad and *ω*_3_=0.421 Hz; cluster 4 (yellow): 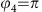 and *ω*_4_=0.440 Hz; forcing at Ω=0.413 Hz.

Uniquely attractive phase patterns are desired, where a common input synchronizes the oscillators to a pattern independently of their initial phases. The fixed point 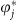 of equation (2) must be unique for each *j*, which is achieved when the interaction function crosses each horizontal line Δ*ω*_*j*_ from above only once at 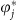. This is possible when the phase offsets are monotonically ordered as 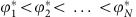 for *ω*_1_<*ω*_2_<…<*ω*_*N*_, as demonstrated by segregation of 20 inhomogeneous electrochemical oscillators into clusters in the experiments described in Fig. 2. An anti-phase configuration 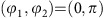 is achieved for electrodes in balanced (*N*_1_, *N*_2_)=(10, 10) and unbalanced (*N*_1_, *N*_2_)=(1, 19) clusters in Fig. 2a,b, respectively. In these two-cluster examples, the interaction function decreases in two steps, of which the top and bottom correspond to clusters with slower (blue) and faster (red) natural frequencies. Figure 2c shows the formation of four balanced clusters of (*N*_1_, *N*_2_, *N*_3_, *N*_4_)=(5, 5, 5, 5) oscillators with the phase structure 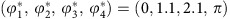 radians. The phase offsets 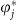 are increasing as −Δ*ω*_*j*_ decreases (and *ω*_*j*_ increases), and the designed interaction function decreases monotonically as it crosses the required intersections. Observe that the assumption of a common PRC is reasonable, because the functions in Fig. 1a are very similar, yet the oscillator frequencies are sufficiently heterogeneous for our technique to create the patterns in Fig. 2.

### Control of pattern transitions in an ensemble

**Figure 3:**
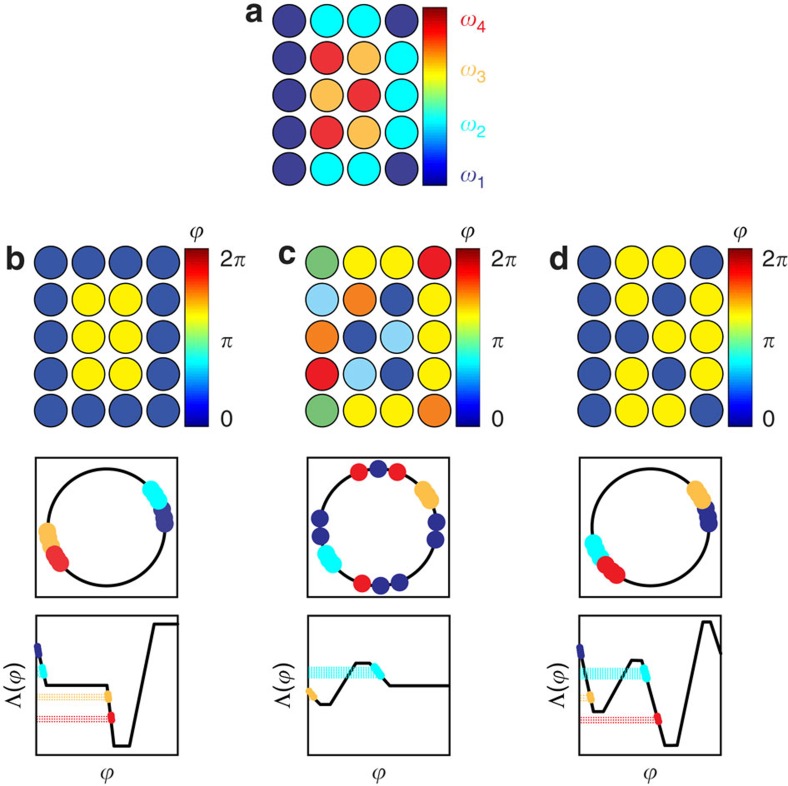
Schematic of pattern switching ‘O→K→O' by phase-selective entrainment. (**a**) Natural frequencies for four spatially distributed oscillator clusters of sizes (*N*_1_, *N*_2_, *N*_3_, *N*_4_)=(7, 7, 3, 3) in an ascending order: *ω*_1_<*ω*_2_<*ω*_3_<*ω*_4_. Cluster 1 contains electrodes *n*=1 to 7 (blue), cluster 2 contains *n*=8 to 14 (cyan), cluster 3 contains *n*=15 to 17 (yellow) and cluster 4 contains *n*=18 to 20 (red). Items **b** to **d** depict snapshots of phase patterns at the forcing phase *θ*=0 (top row), the description of the phases on a unit circle (middle row), and a sketch of the interaction function (bottom row). Phase assignment for: (**b**): Pattern ‘O': 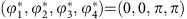. (**c**): Precursor of pattern ‘K': 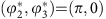. (**d**): Pattern ‘K': 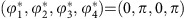.

In addition, we establish patterns without monotone phase ordering by designing an interaction function of the form at the bottom of Fig. 3d, which crosses a horizontal line at −Δ*ω*_*j*_ from above twice, yielding multiple possible entrained phases and dependence on initial conditions. We apply precursor waveforms to steer subsets of the ensemble into attractive regions for the desired phase offsets 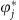, then finalize and hold the pattern with an ultimate input. This procedure is applied to steer an ensemble between spatially associated clusters by alternating selective inputs. Fig. 3 illustrates input design for itinerant formation of letters in the word ‘OK' in the array of 20 electrochemical oscillators used in the experiments in Fig. 2. We produce anti-phase assignment between clusters to display the letter ‘O', then switch the input to produce the letter ‘K'. Rhythmic elements are assigned desired phase offsets of 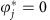 or 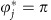, which correspond to ‘on' (pattern) or ‘off' (background) states, respectively, that are visualized using a colour scale where 0 (‘on') is blue and *π* (‘off') is yellow. Switching between two patterns is accomplished using four numbered clusters, where 1 is ‘on' for both, 2 switches from ‘on' to ‘off', 3 switches from ‘off' to ‘on', and 4 is always ‘off'. Electrodes in clusters of (*N*_1_, *N*_2_, *N*_3_, *N*_4_)=(7, 7, 3, 3) elements with mean natural frequencies (*ω*_1_, *ω*_2_, *ω*_3_, *ω*_4_)=(0.390, 0.406, 0.427, 0.442) Hz are positioned in the spatial arrangement in Fig. 3a. Figure 3b–d each have panels that show, from top to bottom, the spatial distribution of phase offsets, the structure on the unit circle, and a sketch of the ideal interaction function.

The pattern ‘O', shown in Fig. 3b, is realized using the phases 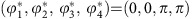, which are achieved by an interaction function as in Fig. 2a. The phases 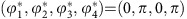 used for ‘K' are not monotonically ordered, so a precursor waveform is applied to generate globally attractive phase offsets 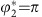 and 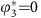 for clusters 2 and 3. This anti-phase pattern establishes initial conditions for the final input waveform, while clusters 1 and 4 lose their entrainment, as shown in Fig. 3c. Figure 3d illustrates the design of the finalizing control for pattern ‘K', where the phase assignments for clusters 1 and 4 are globally attractive, as seen in the bottom panel of Fig. 3d, while clusters 2 and 3 stay at phase offsets established in the precursor stage. The transition from pattern ‘K' back to ‘O' is accomplished by applying the initial control for the pattern in Fig. 3b. We provide additional descriptions of interaction function construction (Supplementary Note 4; Supplementary Figs 1 and 2), pattern realizability (Supplementary Note 5), control design for monotonically ordered patterns (Supplementary Note 6; Supplementary Fig. 3), and precursor waveform engineering (Supplementary Note 7; Supplementary Figs 2 and 4).

**Figure 4:**
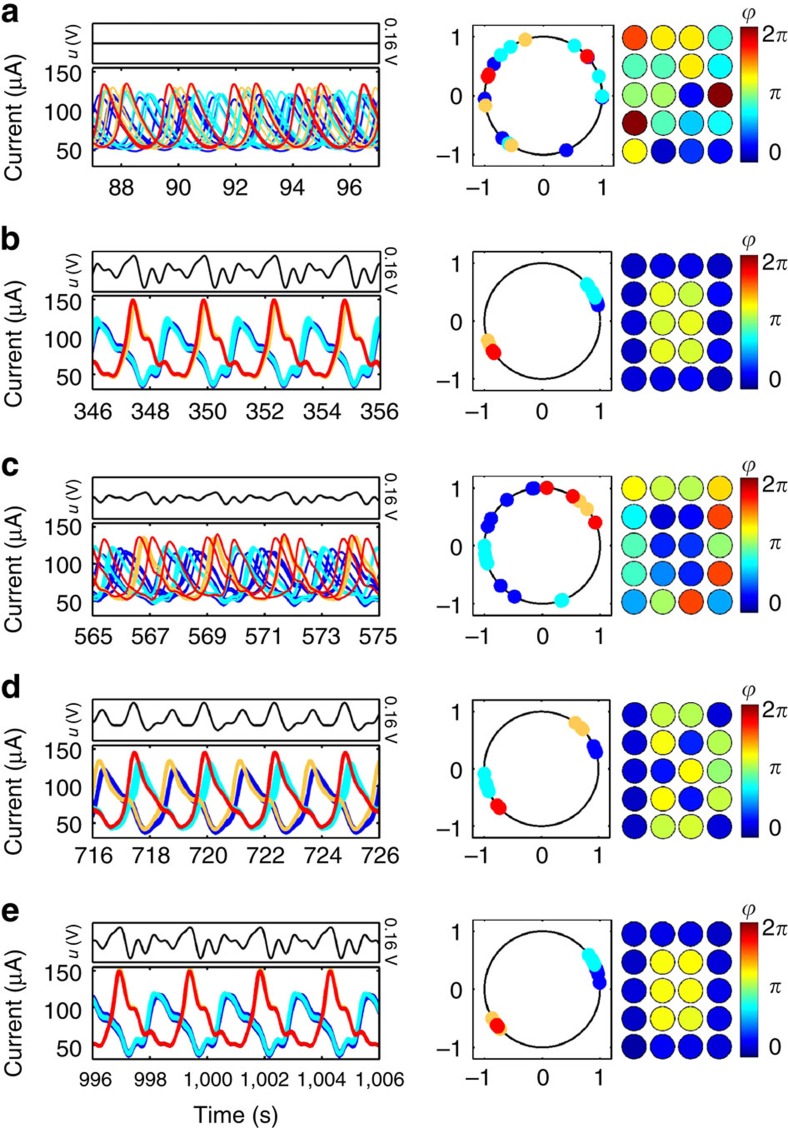
Pattern switching experiment ‘O→K→O' on an array of electrochemical oscillators. Left: current oscillations with the control signal above. Right: oscillator phases on a unit circle and a snapshot of spatial phase assignment at a time *t*_*s*_. (**a**): Current oscillations with no control and mean natural frequencies for four electrode clusters (*N*_1_, *N*_2_, *N*_3_, *N*_4_)=(7, 7, 3, 3) of (*ω*_1_, *ω*_2_, *ω*_3_, *ω*_4_)=(0.390, 0.406, 0.427, 0.442) Hz at *t*_s_=92 s. Phase assignment with forcing at Ω=0.408 Hz for: (**b**): Pattern ‘O': 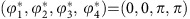, at *t*_s_=351 s. (**c**): Precursor of pattern ‘K': 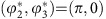, at *t*_*s*_=570 s. (**d**): Pattern ‘K': 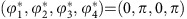, at *t*_*s*_=721 s. (**e**): Same as item (**b**) with *t*_s_=1,001 s.

Measurements of the ‘O→K→O' pattern switching experiment appear in Fig. 4. Figure 4a shows current oscillations for the reaction units given zero input, when no pattern forms. When the controls (shown above the current) are applied, the ensemble is entrained within several cycles, selectively forming the patterns for ‘O', the precursor, and ‘K'. These results are shown in Fig. 4b–d, and correspond to Fig. 3b–d, respectively. The ensemble is returned to pattern ‘O', as shown in Fig. 4e, to demonstrate switching. The automatically generated interaction functions and control waveforms are presented in section ‘Automatic control waveforms generated in experiments'. An animation produced using the experimental current traces and oscillation phases is included as Supplementary Movie 1.

## Discussion

Phase-selective entrainment enables the use of a single global signal to robustly assign elements of a noisy nonlinear oscillator ensemble to specific phases without using coupling or feedback information. Control design using interaction functions simplifies the creation of complex synchronization patterns to drawing or automatically generating curves through sets of crossing points and computing the resulting controls with a simple formula, which is an accessible technique for experimentalists. Greater relaxation in the oscillation and ensemble heterogeneity increases pattern controllability, and performance is improved as the oscillations move farther away from the Hopf bifurcation. The asymptotic nature of entrainment yields robustness to noise, disturbances and model parameter variability while preserving the intrinsic nature of the ensemble.

Such resilience is required for nondestructive control of underactuated, noisy and uncertain biological and chemical ensembles that cannot be readily observed. For example, an effective technology for neurological treatment of Parkinson's disease^17^ is provided by deep brain stimulation, which alleviates pathological synchronization in the brain. Selective entrainment could be extended to ensembles with weak coupling to design robust desynchronization inputs, which would potentially benefit noninvasive neurostimulation technology^41^. The goal could be a target distribution that is found to be optimal for leveraging neuroplasticity to prevent resynchronization after the stimulus is ended. The technique could also improve phase regulation to treat cardiac arrhythmias^16^ and sleep irregularities^45^. The formalism could also represent the entrainment that occurs in circadian timekeeping^14^.

We note that a simple sinusoidal forcing of the form *v*(Ω*t*)=sin (Ω*t*) results in a sinusoidal interaction function, because of orthogonality of the trigonometric Fourier basis. Sinusoidal forcing can thus be used to create monotone ordered phase patterns, and could also be used for desynchronization. However, because such an interaction function is decreasing on an interval of length *π*, the maximum achievable distance between extremal phase offsets 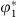 and 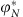 is 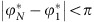. Thus, a sinusoidal input cannot produce anti-phase synchronization. Our approach enables more versatile manipulation of phase relationships beyond this limitation. We describe the application to desynchronization in Supplementary Note 8 and Supplementary Fig. 5, and quantify how our approach increases the achievable relative phase desynchronization difference over sinusoidal forcing. More rigorous mathematical characterization of ensemble desynchronization by periodic inputs is a compelling direction for further investigation.

In our methodology, we take advantage of approximations that are possible in the specific experimental setting. In our experiments, the distribution of natural frequencies of ensemble oscillations varies by ±20% from the (non-zero) mean, the oscillators are very weakly coupled, the amplitude of the required external forcing signal is relatively small, and the entrainment process is approximated well by averaged phase models. In addition, although the ensemble subsystems are slightly heterogeneous and noisy, with variation in natural frequencies and dynamic properties, the phase response curves are very similar. We expect the methodology to function well in other experimental settings in which these conditions are satisfied. Moreover, the method holds promise for extension to other scenarios, e.g., in sub- and superhamornic entrainments (the oscillations are locked to different frequency ratios), where the interaction function-based phase description is possible.

The arrangement of frequency clusters in an oscillator ensemble can also be viewed as encoding information within the spatial pattern produced when selective entrainment is applied. One of several encoded messages can then be retrieved using the phase-selective entrainment process, for which the passkey for retrieving the message is the temporal information contained in a periodic input signal. The passkey is constructed using the PRC and natural frequencies of the dynamical subsystems, and after that input signal is applied, the spatial phase pattern produced in the ensemble reveals the message. This approach may be incorporated in neurocomputing architectures^46^ that mimic neural systems in nature. Future investigation is required to understand how network coupling could be suppressed or taken advantage of to improve pattern resilience and information capacity, or effectively encrypt the message by preventing estimation of PRCs and natural frequencies of oscillators in the spatial array.

## Methods

### Experimental apparatus

**Figure 5:**
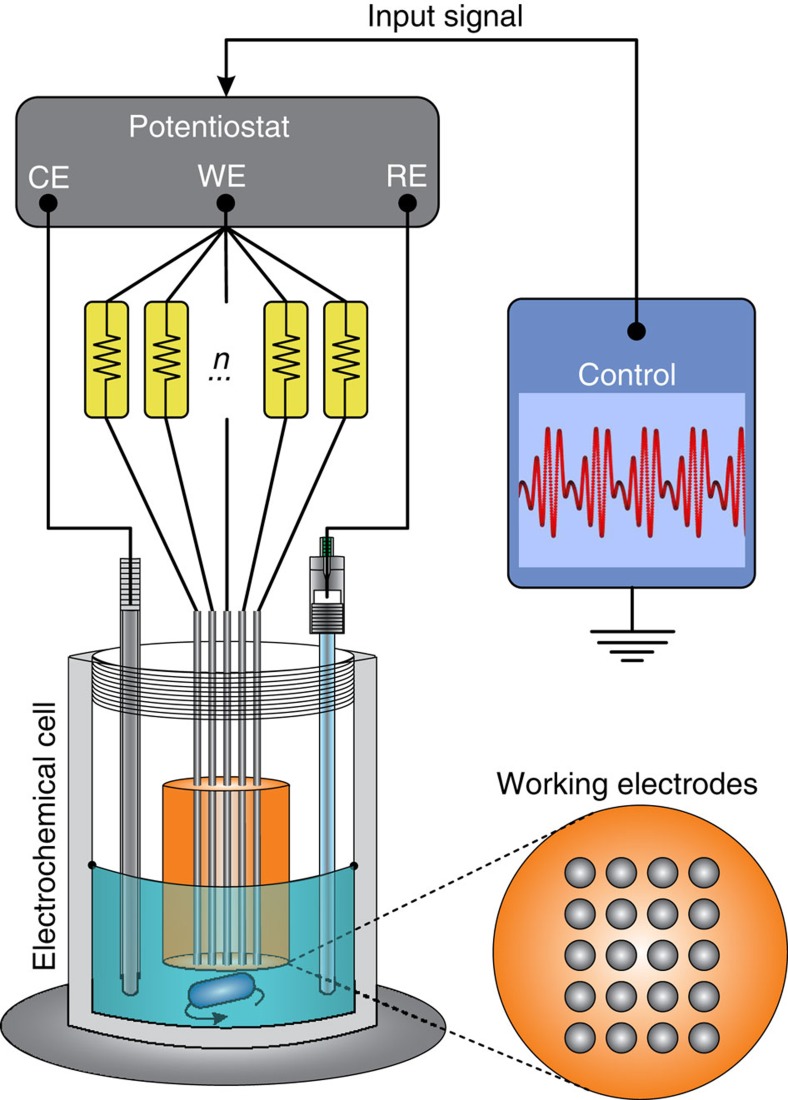
Electrochemical set-up. The working electrode (WE) is composed of an array of nickel wires, the counter electrode (CE) is a Pt coated Ti wire, and the reference electrode (RE) is Hg/Hg_2_SO_4_/sat. K_2_SO_4_. The control waveform is a potential signal generated by the multifunction M Series DAQ PXI-6255 (National Instruments) and implemented in the WE by a potentiostat (Bank Instruments) as a superimposed signal on the applied baseline potential *V*_0_. Each nickel wire is connected in series to an individual resistance *R*_ind_=2,500 Ohm.

Our methodology for controlling the phase structure of an ensemble of heterogeneous oscillators is experimentally verified in the electrochemical dissolution of nickel in 3 mol l^−1^ sulfuric acid solution using potential actuation. A schematic description of the experimental set-up is depicted in Fig. 5. The apparatus consists of 20 nickel wires that function as working electrodes (WE), with diameters of 0.69 mm, spaced by 2.0 mm and embedded in epoxy resin. Prior to the electrochemical measurements, the WE were polished with sandpaper in six levels of roughness, ranging from 180 to 4,000 grit. The current of all electrodes is monitored independently. Under such conditions, the spontaneous formation of self-sustained electrochemical oscillations driven by the presence of a negative differential resistance^47^ is observed in the anodic dissolution of nickel.

Once the WE are placed in the electrochemical cell, a slow positive sweep of 0.01 V s^−1^ from 0 V to a constantly applied potential *V*_0_ was conducted to form a thin passive layer on the electrode surface. This baseline is set in reference to an Hg/Hg_2_SO_4_/sat. K_2_SO_4_ reference electrode (RE) in an electrochemical cell, containing a 1.6 mm diameter Pt coated Ti wire counter electrode at constant temperature of 10 °C. The potential *V*_0_ was initially set using a potentiostat (Bank Instruments) at a value for which the oscillation is close to the Hopf bifurcation, which is ∼1.15 V. Inputs to the oscillating system consist of an additional potential *u* superimposed onto the baseline potential *V*_0_ using the potentiostat.

Soon after, the PRCs were measured simultaneously for the WE by the automatic procedure of applying a pseudorandomly timed potential pulse sequence of −0.20 V with pulse-width of 0.05 s and post-processing the observed current using the pulse perturbation procedure as described in section ‘Entrainment of an ensemble'. Measurements of the current oscillation in the electrochemical reactions were carried out by a real-time data acquisition by a high-speed multifunction M Series DAQ PXI-6255 (National Instruments) interface with a sample rate of 200 Hz. Simultaneously, the periodic potential perturbations *u* were applied in the electrochemical oscillator ensemble using the potentiostat to superimpose the waveform onto the applied baseline voltage *V*_0_. Each control waveform was generated based on the PRC obtained preliminary to the experiment and the targeted interaction function generated from the desired set of phase offsets for the oscillators.

### Automatic control waveforms generated in experiments

**Figure 6:**
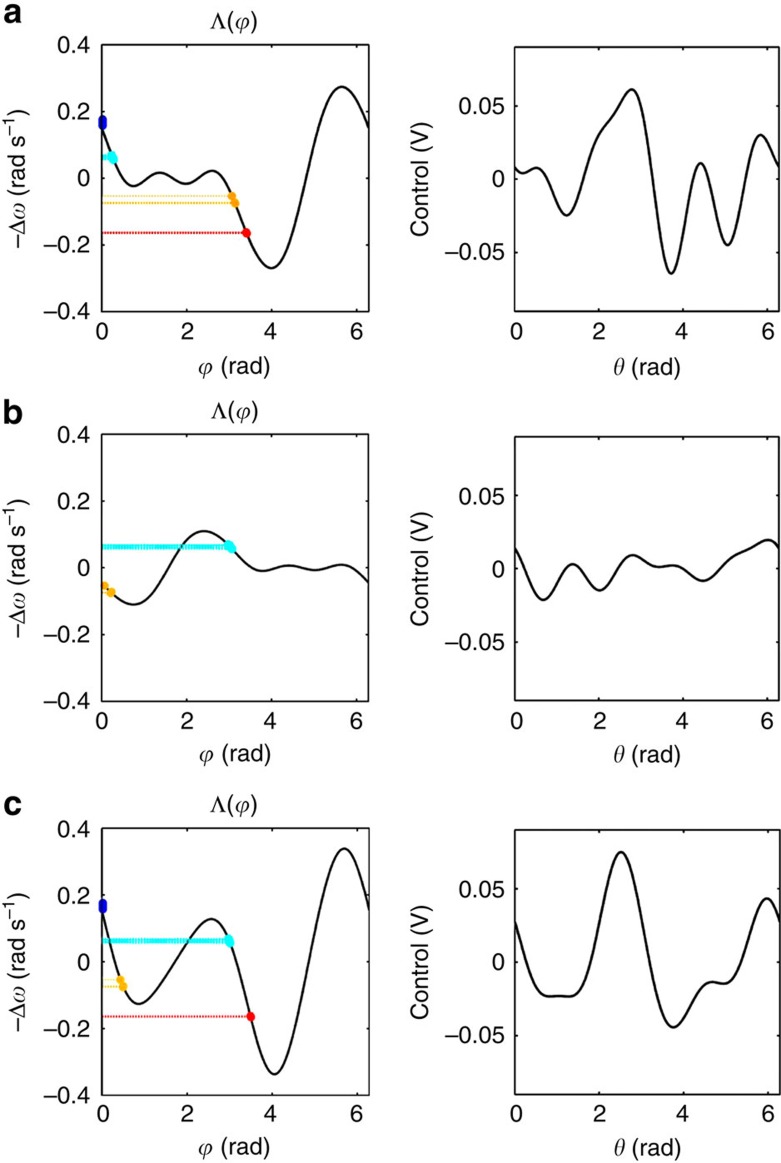
Interaction function and control waveform for pattern switching ‘O→K→O' described in Fig. 4. Left: the best approximation to the desired interaction function; Right: one cycle of the resulting control waveform, obtained using the control design procedure described in section ‘Control of pattern transitions in an ensemble', which is applied to entrain the electrochemical oscillators. The ensemble is grouped into four clusters of quantities (*N*_1_, *N*_2_, *N*_3_, *N*_4_)=(7, 7, 3, 3). Cluster 1 for oscillators *j*=1 to 7 (blue), cluster 2 for *j*=8 to 14 (cyan), cluster 3 for *j*=15 to 17 (yellow) and cluster 4 for *j*=18 to 20 (red). Phase assignment for: (**a**): Pattern ‘O': 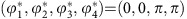. (**b**): Precursor of pattern ‘K': 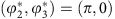. (**c**): Pattern ‘K': 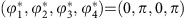.

Figure 6 displays the interaction function (left column) and the respective control waveform (right column) used to entrain the electrochemical oscillators for the pattern switching procedure ‘O→K→O' described in Fig. 4 (see main text for additional details). An electrochemical oscillator ensembles with clusters of size (*N*_1_, *N*_2_, *N*_3_, *N*_4_)=(7, 7, 3, 3) were selected by tuning the mean natural frequency of the oscillations in the following order: cluster 1 with *ω*_1_=0.390 Hz (electrodes *j*=1 to 7 in blue), cluster 2 with *ω*_2_=0.406 Hz (electrodes *j*=8 to 14 in cyan), cluster 3 with *ω*_3_=0.427 Hz (electrodes *j*=15 to 17 in yellow) and cluster 4 with *ω*_4_=0.442 Hz (electrodes *j*=18 to 20 in red). The phase assignments for pattern switching ‘O→K→O' are listed in Fig. 6.

## Additional information

**How to cite this article**: Zlotnik, A. *et al*. Phase-selective entrainment of nonlinear oscillator ensembles. *Nat. Commun.* 7:10788 doi: 10.1038/ncomms10788 (2016).

## Supplementary Material

Supplementary Information Supplementary Figures 1-5, Supplementary Notes 1-8 and Supplementary References.

Supplementary Movie 1 This animation demonstrates pattern formation in an oscillating chemical reaction on a multi-electrode array. The image in the circle at Left illustrates the phase of each oscillator using color as well as a dot on the unit circle. The plots at Right show the control applied to the entire array (top) and the measured current traces (bottom). Initially, when no control is applied, there is no pattern formed. A control is then applied to form the pattern "O", in which phases of oscillators forming the letter are synchronized anti-phase with respect to the others. The control is subsequently modified to form the pattern "K", and finally the initial control is repeated to revert to the pattern "O".
