# Supplementary material for: Phase-selective entrainment of nonlinear oscillator ensembles
Source: Nat Commun. 2016 Mar 18;7:10788. doi: 10.1038/ncomms10788 (PMC4802046; doi:10.1038/ncomms10788)
Supplement: Supplementary Information — Supplementary Figures 1-5, Supplementary Notes 1-8 and Supplementary References. [file ncomms10788-s1.pdf]

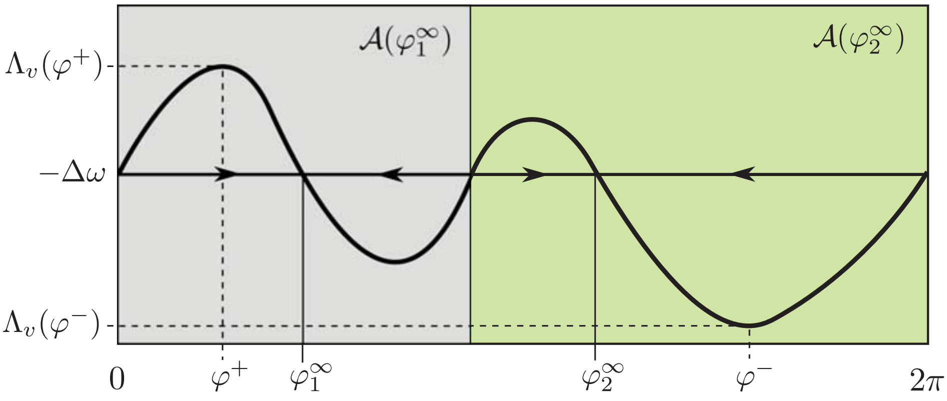

Supplementary Figure 1. Important properties of an interaction function $\Lambda_v(\varphi)$. The maximum and minimum values $\Lambda_v(\varphi^+)$ and $\Lambda_v(\varphi^-)$, which occur at the phases φ^+ and φ^-, respectively, determine the range of frequency detuning for which the oscillator can be entrained using weak forcing. The solutions φ_1^∞ and φ_2^∞ of the equation $\Lambda_v(\varphi) = -\Delta\omega$ that satisfy $\Lambda'(\varphi) < 0$ determine the average phase shift, relative to Ωt, at which the oscillation stabilizes from a given initial phase. Initial phases in the regions $\mathcal{A}(\varphi_1^\infty)$ (grey) and $\mathcal{A}(\varphi_2^\infty)$ (green) result in asymptotic phase shifts of φ_1^∞ and φ_2^∞, respectively. When $\Lambda_v(\varphi) > -\Delta\omega$ then $\dot{\varphi} > 0$ so the phase increases, and when $\Lambda_v(\varphi) < -\Delta\omega$ then $\dot{\varphi} < 0$ so the phase decreases, as indicated by the arrows to illustrate equation (10).

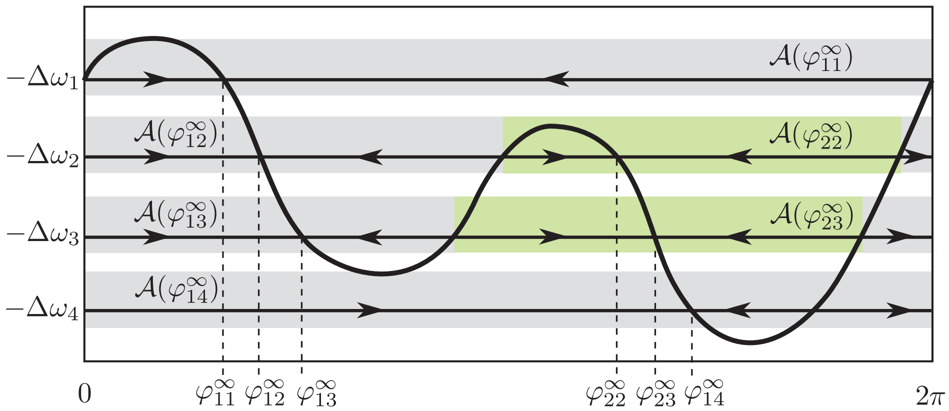

Supplementary Figure 2. Interaction function $\Lambda_v(\varphi)$ that satisfies the conditions (13)-(14) with φ_{11}^∞ for $j = 1$, φ_{12}^∞ or φ_{22}^∞ for $j = 2$, φ_{13}^∞ or φ_{23}^∞ for $j = 3$, and φ_{14}^∞ for $j = 4$. The attractive regions $\mathcal{A}(\varphi_{ij}^\infty)$ are indicated in grey for $i = 1$ and green for $i = 2$.

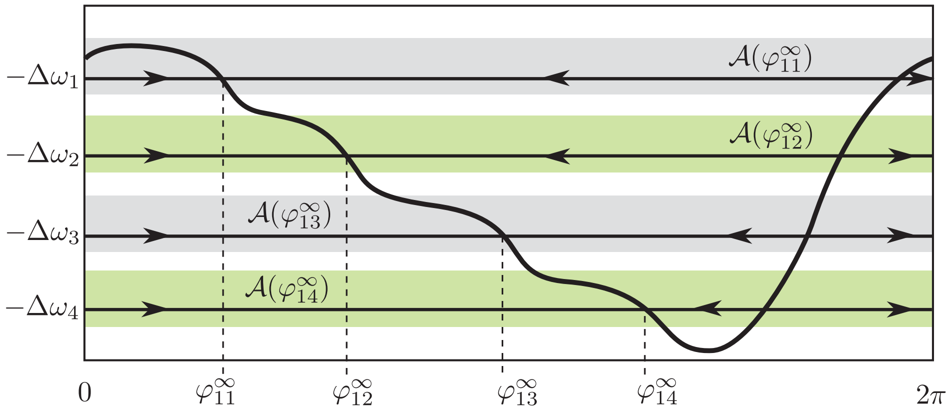

Supplementary Figure 3. Interaction function $\Lambda_v(\varphi)$ designed using the monotonicity method that satisfies the conditions in equations (13)-(14) with a globally attractive phase pattern. The unique fixed points for each oscillator are φ_{11}^{∞}, φ_{12}^{∞}, φ_{13}^{∞}, and φ_{14}^{∞} for $j = 1, 2, 3, 4$, respectively. The attractive regions are $\mathcal{A}_j(\varphi_{1j}^{\infty}) = [0, 2\pi)$ for all j, as indicated.

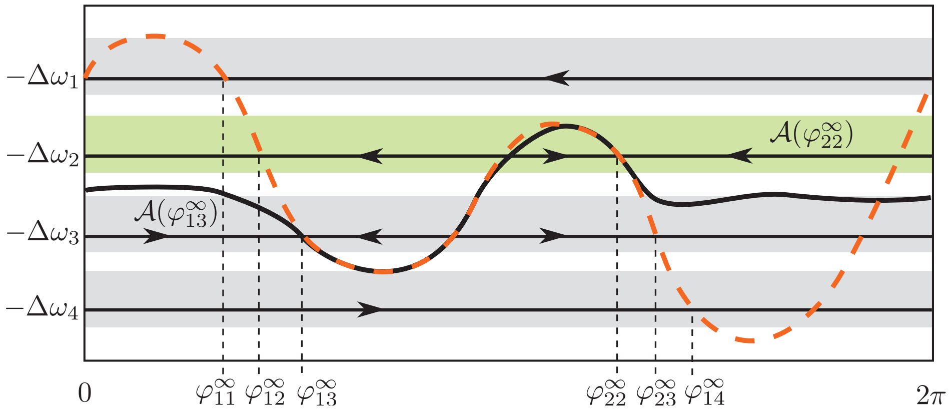

Supplementary Figure 4. Interaction function $\Lambda_v(\varphi)$ corresponding to a precursor input that entrains oscillators $j = 2$ and 3 to the desired phase offsets $\varphi_2^* = \varphi_{22}^{\infty}$ and $\varphi_3^* = \varphi_{13}^{\infty}$. The attractive regions $\mathcal{A}_2(\varphi_{22}^{\infty}) = [0, 2\pi)$ and $\mathcal{A}_3(\varphi_{12}^{\infty}) = [0, 2\pi)$ are global. The waveform generated from the interaction function in Figure B repeated here as an orange dashed line, can be subsequently applied to finalize the pattern.

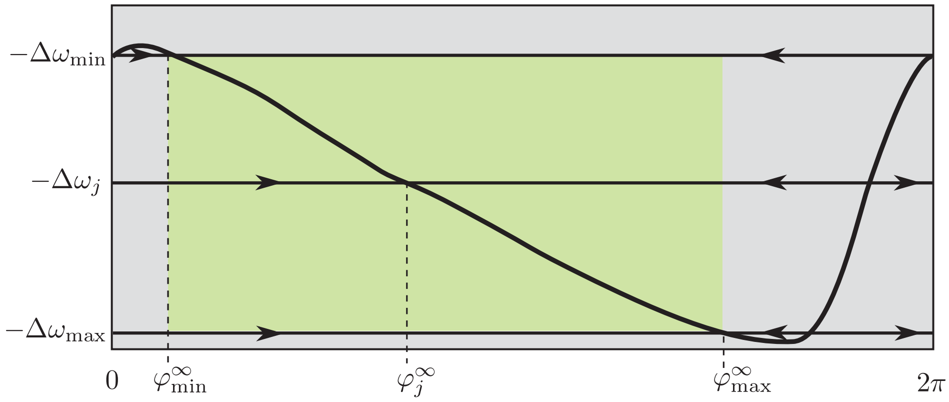

Supplementary Figure 5. Interaction function $\Lambda_v(\varphi)$ corresponding to desynchronization of a large ensemble of oscillators with frequencies distributed uniformly on the interval $[\omega_{\min}, \omega_{\max}]$. The frequency detuning values are distributed uniformly on $[-\Delta\omega_{\max}, \Delta\omega_{\min}]$, so that the control generated from $\Lambda_v(\varphi)$ will disperse the phase offsets uniformly on the interval $[\varphi_{\min}^{\infty}, \varphi_{\max}^{\infty}]$. The asymptotic offset φ_j^{∞} for an oscillator with an intermediate frequency $\omega_j \in [\omega_{\min}, \omega_{\max}]$ is indicated.

Supplementary Note 1: Phase Coordinate Transformation

The technique for phase coordinate transformation stems from the early work of Andronov and Vitt [1], and can be derived directly from the method of Malkin [2]. We formally define an oscillator as a system of smooth ordinary differential equations

$$\dot{x} = f(x, u), \quad x(0) = x_0, \quad t \in [0, \infty) \tag{1}$$

where $x(t) \in \mathbb{R}^n$ is the state and $u(t) \in \mathbb{R}$ is a control function. The oscillatory property of equation (1) implies the existence of an attractive, non-constant limit cycle $\gamma(t) = \gamma(t+T)$, which is a solution to the unperturbed system $\dot{\gamma} = f(\gamma, 0)$, on the periodic orbit defined by $\Gamma = \{y \in \mathbb{R}^n \ : \ y = \gamma(t) \text{ for } 0 \leq t < T\} \subset \mathbb{R}^n$. The key idea is to define a mapping between $\gamma(t)$ and $\psi(t)$ in a way such that the phase of the unperturbed system advances proportionally to time along the periodic orbit, i.e., $\psi(t) = \omega t$, where $\omega = 2\pi/T$ is the natural frequency of oscillation for the unforced oscillator. The dynamics equation (1) can then be approximated in phase coordinates by a scalar equation

$$\dot{\psi} = \omega + Z(\psi)u, \tag{2}$$

which is called a phase model, where Z is the phase response curve (PRC), also called the infinitesimal PRC or iPRC, which quantifies the asymptotic phase shift due to an infinitesimal perturbative input applied at a given phase on the limit cycle. The phase variable $\psi(t)$ is as-

sociated to the isochronous manifold corresponding to $\gamma(t) \in \Gamma$, from which all undisturbed trajectories asymptotically synchronize [3]. We say that $\psi(t) = 0$ (mod 2π) when an observed variable in the state vector $y = \gamma(t)$ attains its maximum on Γ. The phase model equation (2) accurately represents the dynamics equation (1) for sufficiently weak inputs $u(t)$ such that the solution $x(t, x_0, u)$ to equation (1) remains within a small enough neighborhood of Γ [4]. The PRC for a system with known dynamics of the form in equation (1) can be computed by various procedures [5, 6, 7, 8, 9], including an approach similar to that of Malkin that is described in Appendix C of [10].

Supplementary Note 2: Model Identification for Oscillating Systems

Several approaches to model identification have been used to estimate the PRC in real oscillating systems subject to noise and disturbances [11, 12]. We describe our technique for phase model identification, which has been applied previously in experiments on electrochemical systems [13]. This model identification procedure does not require online state observations or feedback, but rather relies on post-processing of a pseudo-random input sequence and observations of the effect on a state variable. Suppose that a brief impulse of duration Δt and magnitude M is applied to an oscillator at time t_0, when the phase is $\psi(t_0)$. Specifically, suppose $u(t) = M$ for $t \in [t_0, t_0 + \Delta t]$, and $u(t) = 0$ elsewhere. Let $\psi_1(t_0 + NT)$ and $\psi_0(t_0 + NT)$ represent the phase value when the oscillator has completed N cycles after the pulse is applied, and in the absence of a pulse, respectively. Because Γ is strongly attractive, the system will relax back to the periodic orbit several cycles after the pulse is applied. Assuming that the pulse duration Δt is brief, $Z(\psi(t))$ is approximately constant on $t \in [t_0, t_0 + \Delta t]$, so that integrating equation (2) results in

$$\psi_0(t_0 + NT) = \omega NT + \psi(t_0), \tag{3}$$

$$\begin{aligned} \psi_1(t_0 + NT) &= \omega NT + \psi(t_0) + \int_{t_0}^{t_0+\Delta t} Z(\psi(t)) M \mathrm{d}t \\ &\approx \omega NT + \psi(t_0) + Z(\psi(t_0)) M \Delta t. \end{aligned} \tag{4}$$

Subtracting equation (3) from equation (4) and solving for $Z(\psi(t_0))$ results in

$$Z(\psi(t_0)) = \frac{\psi_1(t_0 + NT) - \psi_0(t_0 + NT)}{M \Delta t}. \tag{5}$$

The phases ψ_0 and ψ_1 are valued at 0 (equivalently 2π) at peaks of the observed state variable, and their values between peaks are reasonably approximated by linear interpolation [14]. If $y(t)$ is the observed oscillator output, let T_{-1} and T_N denote the latest time at which $y(t)$ reaches a peak before time t_0, and the time when $y(t)$ reaches the Nth peak after the pulse is applied. Because phase on the limit cycle is linearly proportional to time, i.e., $\psi(t) = \omega t$, the phases at two distinct time points $t_1, t_2 \in [0, T)$ will satisfy $\psi(t_1) - \psi(t_2) = \omega \cdot (t_1 - t_2)$. As a result, the

linear interpolation yields

$$\begin{aligned}\psi_1(t_0+NT)-\psi_0(t_0+NT) &\approx \omega\cdot(T_N-T_{-1})-\omega\cdot NT\\ &= 2\pi\cdot\frac{(T_N-T_{-1})-NT}{T},\end{aligned} \tag{6}$$

where N is greater than the sufficient number of limit cycles for the perturbed system ψ_1 to have relaxed to the periodic orbit Γ. In our implementation, we create a sequence of pulses at intervals of $T(N+r_j)$, where $r_j \in (0,1)$ are pseudo-random, so that post-processing results in a time series $Z(\psi_j)$ where $\psi_j = \psi_{j-1} + \omega T(N+r_j)$ (mod 2π). The pulse sequence is applied, and the measured peak times are used to estimate the phase differences in equation (6) in a postprocessing step. The data are fitted using a Fourier series to estimate the PRC $Z(\psi)$. One important advantage of this approach is to enable the concurrent identification of the PRCs for a large ensemble of rhythmic systems with simultaneously measurable output by using a single globally applied pulse sequence applied to the entire ensemble. The measurement state variable signal for each oscillator can then subsequently be processed off-line to produce a collection of phase models. This procedure is used to estimate the PRCs of each electrochemical oscillator in our experimental array, which are shown in Figure 1A of the main text.

Supplementary Note 3: Ergodic Averaging for Oscillating Systems

The central mechanism of our control methodology is entrainment, which refers to the dynamic synchronization of an oscillating system to a periodic input. The properties that characterize entrainment of the system in equation (2) to a periodic forcing control $u(t) = v(\Omega t)$, where v is 2π-periodic and Ω is the forcing frequency, are closely approximated by ergodic averaging when the forcing signal is weak, i.e., $v = \varepsilon v_1$ where v_1 has unit energy and $\varepsilon \ll 1$. Given such an input, the system in equation (1) is guaranteed to remain in a neighborhood of Γ in which the phase model in equation (2) remains valid [4]. A slow phase variable is defined by $\phi(t) = \psi(t) - \Omega t$, and the difference $\Delta\omega = \omega - \Omega$ is called the frequency detuning. The dynamic equation for the slow phase is then

$$\dot{\phi} = \dot{\psi} - \Omega = \Delta\omega + Z(\Omega t + \phi)v(\Omega t), \tag{7}$$

where $\dot{\phi}$ is called the phase drift. The asymptotic behavior of equation (7) is obtained by ergodic averaging, which eliminates the explicit dependence on time on the right hand side [15, 16]. We define an averaging operator $\langle\cdot\rangle : \mathcal{P} \to \mathbb{R}$, where $\mathcal{P}$ is the set of 2π-periodic functions on $\mathbb{R}$, by

$$\langle x\rangle = \frac{1}{2\pi}\int_0^{2\pi} x(\theta)\mathrm{d}\theta. \tag{8}$$

Then the weak ergodic theorem for measure-preserving dynamical systems on the torus [17] implies that for any periodic function v, the interaction function

$$\begin{aligned}\Lambda_v(\phi) &\triangleq \langle Z(\theta+\phi)v(\theta)\rangle \\ &= \frac{1}{2\pi}\int_0^{2\pi} Z(\theta+\phi)v(\theta)\mathrm{d}\theta \\ &= \lim_{T\to\infty}\frac{1}{T}\int_0^T Z(\Omega t+\phi)v(\Omega t)\mathrm{d}t \end{aligned} \tag{9}$$

exists as a continuous, 2π-periodic function in $\mathcal{P}$, where $\theta = \Omega t$ is called the forcing phase.

The formal averaging theorem [16] permits us to approximate equation (7) by the averaged system

$$\dot{\varphi} = \Delta\omega + \Lambda_v(\varphi), \tag{10}$$

in the sense that there exists a change of variables $\varphi = \phi + \varepsilon h(\varphi, \phi)$ that maps solutions of equation (7) to those of equation (10). It has been proved that this approximation is of order $\mathcal{O}(\varepsilon^2)$ (Appendix B of [18]), and hence it is appropriate in the case of weak forcing ($v = \varepsilon v_1$ with $\varepsilon \ll 1$). The averaged equation (10) is autonomous, and approximately characterizes the asymptotic behavior of the system in equation (2) under periodic forcing. Specifically, we say that the system is entrained by a control $u = v(\Omega t)$ when the phase drift equation (10) satisfies $\dot{\varphi} = 0$, which will occur as $t \to \infty$ if there exists a phase φ^∞ that satisfies $\Delta\omega + \Lambda_v(\varphi^\infty) = 0$. Conversely, when both the control waveform v and PRC Z are non-zero, the function $\Lambda_v(\varphi)$ is not identically zero, so when the system is entrained there exists at least one $\varphi^\infty \in [0, 2\pi)$ that is an attractive fixed point of equation (10). Such stable fixed points $\{\varphi_i^\infty\}$ of equation (10) are the roots of the equation $\Delta\omega + \Lambda_v(\varphi) = 0$ that also satisfy $\Lambda_v'(\varphi) < 0$, where $\Lambda_v'(\varphi) = \frac{\mathrm{d}}{\mathrm{d}\varphi}\Lambda_v(\varphi)$. The values $\{\varphi_i^\infty\}$ determine the average phase shift, relative to the forcing phase $\theta = \Omega t$, at which the oscillation stabilizes from an initial phase difference $\varphi(0)$. We denote by $\mathcal{A}(\varphi_i^\infty) \subset [0, 2\pi)$ the set of initial phases $\varphi(0)$ that result in convergence of the oscillator to φ_i^∞. In addition, we define the phases $\varphi^+ = \arg\max_\varphi \Lambda_v(\varphi)$ and $\varphi^- = \arg\min_\varphi \Lambda_v(\varphi)$ at which the interaction function achieves its maximum and minimum values, respectively. In order for entrainment to occur, $-\Lambda_v(\varphi^+) \leq \Delta\omega \leq -\Lambda_v(\varphi^-)$ must hold, so that at least one stable fixed point of Λ_v exists. Thus the range of the interaction function determines which values of the frequency detuning $\Delta\omega$ yield phase locking, and the shape of Λ_v determines the possible asymptotic phase shifts φ_i^∞. These properties are illustrated in Supplementary Figure 1.

Supplementary Note 4: Ensemble Interaction Function and Control Input Construction

In this note we examine the interaction function of a structurally similar ensemble and how one can be constructed and processed to create a control input with a desired phase selection effect. We use the interaction function formalism to derive the long-run behavior of a collection of N nonlinear oscillators with phase-reduced dynamics of the form in equation (2) given by

$$\mathcal{F} = \{\dot{\psi}_j = \omega_j + Z(\psi_j)u,\ j = 1, \ldots, N\} \tag{11}$$

which all share an identical PRC. We suppose, without loss of generality, that the natural frequencies of the ensemble elements are ordered according to $\omega_1 < \omega_2 < \ldots < \omega_N$. If an input of the form $u(t) = v(\Omega t)$, where v is 2π-periodic, is applied to all the oscillators in $\mathcal{F}$, we obtain the collection of average phase drift dynamics of the form in equation (10) given by

$$\langle \mathcal{F} \rangle = \{\dot{\varphi}_j = \Delta\omega_j + \Lambda_v(\varphi_j),\ j = 1, \ldots, N\}. \tag{12}$$

If each average drift equation in $\langle \mathcal{F} \rangle$ has a fixed point, then the input waveform $v(\Omega t)$ entrains all of the oscillators in $\mathcal{F}$ to the frequency Ω, and the pattern that emerges in the phase offsets φ_j^∞ of the oscillators relative to the forcing phase $\theta = \Omega t$ can be inferred from the interaction function, the frequencies ω_j, and the initial conditions $\varphi_j(0)$. Specifically, the average phase drift $\dot{\varphi}_j$ of each synchronized oscillator will be zero, and the average phase offset from the forcing phase $\theta = \Omega t$ will be φ_{ij}^∞, depending on which set $\mathcal{A}_j(\varphi_{ij}^\infty)$ the initial phase $\varphi_j(0)$ belongs to. Here $\mathcal{A}_j(\varphi_{ij}^\infty)$ denotes the set of initial phases for which the jth oscillator of $\langle \mathcal{F} \rangle$ is attracted to the asymptotic phase offset φ_{ij}^∞. When synchronization of the collection $\langle \mathcal{F} \rangle$ to the fixed points φ_{ij}^∞ occurs, then $\dot{\varphi}_j = \Delta\omega_j + \Lambda_v(\varphi_j) = 0$ holds, with

$$\Lambda_v(\varphi_{ij}^\infty) = -\Delta\omega_j \quad j = 1, \ldots, N, \tag{13}$$

$$\Lambda_v'(\varphi_{ij}^\infty) < 0, \quad j = 1, \ldots, N, \tag{14}$$

where φ_{ij}^∞ one of the possible asymptotic phases for the jth oscillator. The condition in equation (14) guarantees that the dynamical configuration is locally attractive and stable, as illustrated for an individual oscillator in Supplementary Figure 1. Conversely, if Λ_v satisfies equations (13)-(14), and the initial phases of the oscillators in $\mathcal{F}$ satisfy $\psi_j(0) = \varphi_{ij}^\infty$ for $j = 1, \ldots, N$, then the synchronization pattern will be maintained. The pattern is also established and maintained when the initial conditions are relaxed to $\psi_j(0) \in \mathcal{A}_j(\varphi_{ij}^\infty)$ for $j = 1, \ldots, N$. These regions are illustrated for an example interaction function in Supplementary Figure 2. Note that for a given collection of entrained oscillators in equation (12) the stable synchronization pattern may not be unique, but depends on the initial conditions $\psi_j(0)$ of the collection (11).

The above analysis describes how the interaction function between a forcing waveform v and the PRC Z common to the collection $\mathcal{F}$ characterizes the asymptotic phase structure of an oscillator ensemble with heterogeneous natural frequencies. We can then examine the construc-

tion of ideal interaction functions for desired phase patterns, and the properties of the ensemble that determine their realizability. Because the $Z(\theta)$, $v(\theta)$, and $\Lambda_v(\varphi)$ are all 2π-periodic, they may be expressed using Fourier series, and the Fourier series for $\Lambda_v(\varphi)$ is readily computed using equation (9), trigonometric identities, and the orthogonality of the Fourier basis. We represent the functions Z and v using the truncated series expansions

$$Z(\theta) \approx Z^r(\theta) = \frac{a_0}{2} + \sum_{n=1}^{r}[a_n \cos(n\theta) + b_n \sin(n\theta)], \tag{15}$$

$$v(\theta) \approx v^r(\theta) = \frac{c_0}{2} + \sum_{n=1}^{r}[c_n \cos(n\theta) + d_n \sin(n\theta)], \tag{16}$$

where the appropriate order r will be discussed below. Applying trigonometric angle sum identities to equation (9) and the orthogonality of the Fourier basis to eliminate terms yields

$$\Lambda_v^r(\varphi) = \frac{f_0}{2} + \frac{1}{2}\sum_{n=1}^{r}[f_n \cos(n\varphi) + g_n \sin(n\varphi)], \tag{17}$$

where

$$f_0 = \frac{a_0 c_0}{2}, \quad f_n = a_n c_n + b_n d_n, \quad g_n = b_n c_n - a_n d_n. \tag{18}$$

Therefore given truncated Fourier series expansions $\Lambda_v^r(\theta)$ and $Z^r(\theta)$, the Fourier coefficients of the corresponding truncated series for the control waveform $v^r(\theta)$ are given by

$$c_0 = 2\frac{f_0}{a_0}\chi_{[a_0 \neq 0]}, \quad c_n = 2\frac{f_n a_n + b_n g_n}{a_n^2 + b_n^2}\chi_{[a_n^2+b_n^2 \neq 0]}, \quad d_n = 2\frac{f_n b_n - a_n g_n}{a_n^2 + b_n^2}\chi_{[a_n^2+b_n^2 \neq 0]}, \tag{19}$$

where $\chi_A = 1$ if A is true, and $\chi_A = 0$ otherwise.

An ideal interaction function $\Lambda_v(\varphi)$ that corresponds to the desired phase assignment task in equations (13)-(14) can be designed using a sum of scaled and shifted sigmoid functions. We use the error function, which is given by

$$\operatorname{erf}(x) = \frac{2}{\sqrt{\pi}}\int_0^x e^{-t^2}\mathrm{d}t, \tag{20}$$

as the basic element of our construction of the designed ideal interaction function that passes precisely through the coordinates $(\varphi_j^*, -\Delta\omega_j)$. We first define a smooth approximation to the unit step function by

$$\sigma(x) = \frac{1}{2}(\operatorname{erf}(2x) + 1), \tag{21}$$

which satisfies $\sigma(-1) \approx 0$, $\sigma(1) \approx 1$, and $\sigma(0) = 1/2$. We then create the range points

$$r_1 = -\Delta\omega_1 + \frac{1}{2}(-\Delta\omega_1 + \Delta\omega_2), \quad (22)$$
$$r_j = \tfrac{1}{2}(-\Delta\omega_j - \Delta\omega_{j-1}), \quad j = 2, \ldots, N \quad (23)$$
$$r_{N+1} = -\Delta\omega_N + \frac{1}{2}(-\Delta\omega_{N-1} + \Delta\omega_N), \quad (24)$$

domain points

$$s_1 = \tfrac{1}{2}(\varphi_1^* + \varphi_N^* - 2\pi), \quad (25)$$
$$s_j = \tfrac{1}{2}(\varphi_j^* + \varphi_{j-1}^*), \quad j = 2, \ldots, N \quad (26)$$
$$s_{N+1} = \tfrac{1}{2}(\varphi_1^* + \varphi_N^* + 2\pi), \quad (27)$$

and domain scaling factors

$$h_1 = \min\{s_2 - \varphi_1^*, (\varphi_1^* - s_1)/2\}, \quad (28)$$
$$h_j = \min\{s_{j+1} - \varphi_j^*, \varphi_j^* - s_j\}, \quad j = 2, \ldots, N \quad (29)$$
$$h_{N+1} = \min\{\tfrac{1}{2}(s_{N+1} - \varphi_N^*), \tfrac{1}{2}(\varphi_1^* - s_1)\}. \quad (30)$$

A suitable interaction function Λ_v^* is then constructed by

$$\begin{aligned}\Lambda_v^*(\varphi) &= r_1 + (r_1 - r_{N+1})\sigma((\varphi - s_1)/h_{N+1}) \\ &\quad + \sum_{j=2}^{N}(r_{j+1} - r_j)\sigma((\varphi - \varphi_j^*)/h_j) + \sum_{j=2}^{N}(r_{j+1} + r_j)\sigma((\varphi + 2\pi - \varphi_j^*)/h_j) \\ &\quad + (r_1 - r_{N+1})\sigma((\varphi - s_{N+1})/h_{N+1}). \quad (31)\end{aligned}$$

The function in equation (31) will thus pass close to the phase and detuning pairs $(\varphi_j, -\Delta\omega_j)$. The sums in (31) are repeated with the added offset factors 2π and -2π because the sigmoid function (21) is not a perfect Heaviside unit step, but a periodic interaction function Λ_v^* must nevertheless be created. We note that the particular methodology used to automatically create a suitable interaction function Λ_v^* that approximately satisfies (13)-(14) is not crucial in itself. As long as the designed interaction function satisfies these properties, the Fourier coefficients (19) obtained by circular de-convolution of this interaction function Λ_v^* and the nominal ensemble PRC Z yields the control that produces the desired phase pattern. We provide equations (20)-(31) as a suggested example algorithm, which we have ourselves used for automatic control design for the experiments in the main text. We emphasize that a satisfactory interaction function curve Λ_v^* is not unique, could be constructed using many different algorithms, and the need to satisfy (13)-(14) is the only common requirement. The key idea is the indirect design of control inputs by constructing an interaction function, and this is the concept that we wish to

communicate.

The resulting function Λ_v^* is approximated by a truncated r-order Fourier series Λ_v^r as in equation (17), which is then used to compute the input waveform in equation (19). The maximal order r is determined by the number of square amplitudes A_n^2 of the Fourier series terms in the PRC that exceed a tolerance δ_Z. In order for $\Lambda_v^r(\varphi)$ to satisfy the design conditions in equations (13)-(14), it must sufficiently well approximate $\Lambda_v(\varphi)$, and therefore the realizability of the design in equations (13)-(14) ultimately depends on the complexity of the PRC, in addition to the frequency distribution of the ensemble and the desired phase offsets. When these conditions are satisfied, the control waveform $v^r(\theta)$ that results in the desired phase pattern can be easily synthesized using the Fourier coefficient formula in equation (19).

Supplementary Note 5: Interaction Function Realizability

The nonlinear complexity of the common PRC of oscillating subsystems in an ensemble determines the realizability of a desired interaction function for the ensemble. Specifically, a greater number of significant terms in the Fourier series approximation of the PRC are obtained as the oscillation exhibits higher relaxational behavior. Typically, this occurs as the oscillation moves farther past a Hopf bifurcation.

By inspecting equation (19), it is evident that $v^r(\theta)$ exists for a pair $\Lambda_v^r(\theta)$ and $Z^r(\theta)$ only if $a_n^2 + b_n^2 > 0$ for all n for which $f_n > 0$ or $g_n > 0$, and when $a_0 \neq 0$ if $f_0 \neq 0$. Therefore, any terms of the Fourier series for Λ_v for which these conditions are not satisfied must be removed from the construction. For actual biological and chemical oscillating systems, the magnitudes $A_n^2 = a_n^2 + b_n^2$ decay approximately exponentially. It is thus crucial for the expansion order r to be appropriately chosen such that $A_n^2 > \delta_Z$ for $n \leq r$, where δ_Z is an appropriate tolerance, and any order n for which $A_n = 0$ occurs must be omitted from the truncated series. This guarantees that numerical conditioning errors do not arise, and also that the designed $v^r(\theta)$ satisfies the weak forcing assumption. An appropriate empirical value for the tolerance is $\delta_Z = \langle Z^2 \rangle \cdot 10^{-4}$. Hence given the input $u(t) = v^r(\Omega t)$, the asymptotic configuration of the entrained oscillators of equation (12) will approximately satisfy the conditions in equations (13)-(14).

For the experimental electrochemical oscillators described in the main text, the appropriate series truncation is $r = 5$. This limits the types of phase patterns that can be achieved for ensembles of such oscillators, and in particular, explains why the observed and desired values of $\Delta\varphi$ in the parity diagram in Figure 1B of the main text diverge near $\Delta\varphi = \frac{4}{5} \cdot 2\pi$. The interaction function must be decreasing at $\Lambda_v(\varphi_2^*) = -\Delta\omega_2$, then return and again decrease at $\Lambda_v(\varphi_1^*) = -\Delta\omega_1$. Because only 5 Fourier series terms can be used to synthesize Λ_v, the derivative $\frac{\mathrm{d}}{\mathrm{d}\varphi}\Lambda_v(\varphi)$ is limited when v has limited amplitude, so there is a minimum phase gap required for this return. This capacity for return of Λ_v disappears as $\Delta\varphi$ approaches 2π, as seen in the left panel of Figure 1D of the main text.

The realizability of the interaction function in the above sense corresponds directly to realizability of a particular phase pattern in the ensemble. Thus, the existence of a periodic waveform that entrains an ensemble to such a phase pattern depends on the PRC of the ensemble sub-

elements. In particular, a greater number of significant terms in the Fourier series expansion of Z corresponds to greater flexibility in phase pattern construction. Thus, more highly nonlinear and relaxational oscillation dynamics reflect greater controllability of the ensemble. Further work to more rigorously characterize this property of oscillatory ensembles is compelling.

Supplementary Note 6: Interaction Functions for Globally Attractive Patterns

A control input waveform is globally attractive for a phase pattern if frequency detuning values for the oscillators are monotone decreasing as desired phase offsets increase, and the corresponding interaction function can be realized. Recall that we suppose the natural frequencies of the ensemble $\mathcal{F}$ to be ordered according to $\omega_1 < \omega_2 < \ldots < \omega_N$. Then the frequency detuning values $\Delta\omega_j = \omega_j - \Omega$ in $\langle\mathcal{F}\rangle$ satisfy $-\Delta\omega_1 > -\Delta\omega_2 > \ldots > -\Delta\omega_N$. Suppose also that the desired phase assignment satisfies $\varphi_1^* < \varphi_2^* < \ldots < \varphi_N^*$. Then an ideal interaction function that satisfies the appropriate conditions in equations (13)-(14) can be designed to have a unique set of asymptotic average phase offsets $\varphi_j^\infty = \varphi_j^*$ that are globally attractive, with $\mathcal{A}_j(\varphi_j^*) = [0, 2\pi)$. The desired interaction function is monotone decreasing for phases on $[-\delta_1, \varphi_N^* + \delta_2)$ (mod 2π) and monotone increasing on $(\varphi_N^* + \delta_2, 2\pi - \delta_1)$ to satisfy periodicity, where δ_1 and δ_2 are sufficient gaps for $\frac{\mathrm{d}}{\mathrm{d}\varphi}\Lambda_v(\varphi)$ to change sign. An example of such a Λ_v, which is of the same form as those used in the experiments shown in Figure 2 of the main text, is illustrated in Supplementary Figure 3.

Application of the waveform v that produces the interaction function Λ_v in Supplementary Figure 3 through the circular convolution in equation (9) will asymptotically guide a rhythmic ensemble of four oscillators into a coherent phase configuration with phase offsets φ_{11}^∞, φ_{12}^∞, φ_{13}^∞, and φ_{14}^∞ for oscillators $j = 1, 2, 3, 4$. In essence, we take advantage of the heterogeneity in natural frequencies ω_j and the nonlinearity in Z to design a weak, low-amplitude waveform that achieves and maintains such a synchronization structure without altering the fundamental dynamics of individual rhythmic units. Monotone ordering of the assigned phases in the same order as natural frequencies allows the synthesis of an ideal interaction function that achieves the desired stable fixed-point average phases $\varphi_{1j}^\infty = \lim_{t\to\infty} \varphi_j(t) = \varphi_j^*$. Moreover, such a Λ_v satisfies $\mathcal{A}_j(\varphi_j^*) = [0, 2\pi)$ for all $j = 1, \ldots, N$, so that the pattern will be established for any set of initial conditions $\psi_i(0)$, so that the phase structure is globally attractive. Finally, suppose that the coefficients of the Fourier series of the PRC Z of the oscillators in $\mathcal{F}$ satisfy $A_n^2 > \delta_Z = \langle Z^2\rangle \cdot 10^{-4}$ for all $n < N$, so that the deconvolution of Λ_v as given in equations (15)-(19) may be done using truncated series with $r = N$ terms.

Supplementary Note 7: Precursor Waveforms for Non-Globally Attractive Patterns

If the desired phase pattern is not monotone in the sense described in Supplementary Note 6, then a control waveform does not exist such that the pattern is globally attractive. In that case,

a precursor waveform is required to bring the phases of ensemble elements into an arrangement after which an ultimate waveform is applied to finalize and hold the phase pattern. When the desired phase offsets φ_j^* corresponding to natural frequencies ω_j are not uniformly increasing on $[0, 2\pi)$, a globally attractive phase structure cannot be achieved. In such cases, as the one illustrated in Supplementary Figure 2, one or more precursor inputs must be applied to steer subsets of the ensemble elements into the desired attractive regions $\mathcal{A}_j(\varphi_j^*)$, after which a final input is applied to finalize and subsequently hold the pattern. For the example in Supplementary Figure 2, a precursor waveform can be applied to entrain oscillators $j = 2$ and 3 to designed phase offsets $\varphi_2^* = \varphi_{22}^\infty$ and $\varphi_3^* = \varphi_{13}^\infty$, as shown in Supplementary Figure 4. When this preliminary entrainment step is accomplished with respect to the chosen forcing phase, the control input is changed to the one corresponding to the Λ_v for the full pattern in Supplementary Figure 2, which is outlined using a dashed line in Supplementary Figure 4 as well. This two-step procedure guarantees that the final outcome is the desired phase assignment, rather than one of several possible patterns. This is the situation that characterizes the experiment "O→K→O" as illustrated in Fig. 3 and Fig. 6 in the main text.

Supplementary Note 8: Interaction Function for Desynchronization

Suppose that we wish to accomplish phase desynchronization of a very large ensemble of oscillators $\mathcal{F}$, where N is large but uncertain with the frequencies ω_j distributed uniformly on an interval $[\omega_{\min}, \omega_{\max}]$. The ideal interaction function Λ_v corresponding to a waveform that will spread the phase offsets of the ensemble uniformly across a large domain is shown in Supplementary Figure 5. Note that because the interaction function satisfies the monotonicity property, the desynchronizing effect is global for any initial configuration of the ensemble. The Fourier series coefficients of the desynchronizing control waveform v are then obtained from the formula in equation (19) for circular deconvolution from Λ_v. A natural continuation of our work on generation of coherent phase structures using global controls without feedback pertains to the effect of such controls on networks of weakly coupled oscillators. Specifically, in the presence of weak coupling, the averaged dynamic equation in (12) for the jth oscillator becomes $\dot{\varphi}_j = \Delta\omega_j + \Lambda_v(\varphi_j) + \varepsilon \sum_i H_{ij}(\varphi_j - \varphi_i)$, where H_{ij} characterizes the interaction between the ith and jth oscillators. If the coupling strength is weak relative to the interaction function Λ_v, i.e., $0 < \varepsilon \ll 1$, then the coupling terms will have little influence on the ability to construct phase patterns in the ensemble by external inputs. We may then pose the mathematical problem of characterizing the desynchronization action of this type of control and how it is affected as coupling strength ε increases. A phase transition may occur after a critical coupling strength is reached after which the desynchronizing action is overpowered by synchronization due to coupling. As a result, a continued investigation in the direction of effectively desynchronizing entrainment controls that account for couplings and topology is warranted. In the context of affecting pathologically synchronized neuronal ensembles, progress in this direction will have particularly important implications for research and clinical neuroscience applications.

Consider that a simple sinusoidal forcing of the form $v(\Omega t) = \sin(\Omega t)$ can be used to desyn-

chronize the ensemble with phase offsets on $[0, \pi)$. This is because it results in an interaction function that decreasing on an interval of length π, so the maximum phase difference achievable is $|\varphi_{\max}^{\infty} - \varphi_{\min}^{\infty}| < \pi$. Hence, our approach enables more versatile manipulation of phase relationships beyond this limitation. In fact, it is possible to quantify how our approach increases the achievable relative phase desynchronization difference relative to sinusoidal forcing. If the PRC has non-trivial terms in its Fourier series up to order r, then it is possible to create an input waveform that results in an interaction function that is monotone on the interval $[0, (2 - \frac{1}{r}\pi)]$. Thus, our approach can be used to achieve maximal desynchronization of an oscillator ensemble by increasing the phase offset distribution range by a factor of $2 - \frac{1}{r}$ period.

Supplementary References

[1] A. A. Andronov, S. E. Khaikin, and A. A. Vitt. Teoriya Kolebanii. 1937.

[2] I. Malkin. *Methods of Poincare and Liapunov in the theory of nonlinear oscillations*. Gostexizdat, Moscow, 1949.

[3] J. Guckenheimer. Isochrons and phaseless sets. *Journal of Mathematical Biology*, 1(3):259–273, 1975.

[4] D. Efimov. Phase resetting control based on direct phase response curve. *Journal of mathematical biology*, 63(5):855–879, 2011.

[5] B. Ermentrout. Type I Membranes, Phase Resetting Curves, and Synchrony. *Neural Computation*, 8(5):979–1001, 1996.

[6] B. Ermentrout. *Simulating, Analyzing, and Animating Dynamical Systems: A Guide to XPPAUT for Researchers and Students*. SIAM, 2002.

[7] E. Brown, J. Moehlis, and P. Holmes. On the Phase Reduction and Response Dynamics of Neural Oscillator Populations. *Neural Computation*, 16(4):673–715, 2004.

[8] W. Govaerts and B. Sautois. Computation of the Phase Response Curve: A Direct Numerical Approach. *Neural Computation*, 18(4):817–847, 2006.

[9] A. Guillamon and G. Huguet. A computational and geometric approach to phase resetting curves and surfaces. *SIAM Journal on Applied Dynamical Systems*, 8(3):1005–1042, 2009.

[10] A. Zlotnik and J.-S. Li. Optimal subharmonic entrainment of weakly forced nonlinear oscillators. *SIAM Journal on Applied Dynamical Systems*, 13(4):1654–1693, 2014.

[11] R. F. Galan, G. B. Ermentrout, and N. N. Urban. Efficient estimation of phase-resetting curves in real neurons and its significance for neural-network modeling. *Physical Review Letters*, 94(15):158101, 2005.

[12] I. Z. Kiss, Y. M. Zhai, and J. L. Hudson. Predicting mutual entrainment of oscillators with experiment-based phase models. *Phys. Rev. Lett.*, 94(24):248301, 2005.

[13] A. Zlotnik, Y. Chen, I. Z. Kiss, H.-A. Tanaka, and J.-S. Li. Optimal waveform for fast entrainment of weakly forced nonlinear oscillators. *Phys. Rev. Lett.*, 111:024102, Jul 2013.

[14] A. Pikovsky, M. Rosenblum, and J. Kurths. *Synchronization: A Universal Concept in Nonlinear Science*. Cambridge University Press, 2001.

[15] Y. Kuramoto. *Chemical Oscillations, Waves, and Turbulence*. Springer, New York, 1984.

[16] F. Hoppensteadt and E. Izhikevich. *Weakly connected neural networks*. Springer-Verlag, New Jersey, 1997.

[17] I. Kornfeld, S. Fomin, and Y. Sinai. *Ergodic theory: Differentiable Dynamical Systems*, volume 245 of *Grund. Math. Wissens.* Springer-Verlag, 1982.

[18] A. Zlotnik and J.-S. Li. Optimal entrainment of neural oscillator ensembles. *J. Neural Eng.*, 9(4):046015, 2012.
